# How to improve the rehabilitation outcome after total knee replacement?

**DOI:** 10.1093/eurpub/ckac131.299

**Published:** 2022-10-25

**Authors:** L Dionisi, G De Gironimo, F Praino, G Abinova, C Averame, AM Gentile, N Nante

**Affiliations:** 1 Post Graduate School of Public Health, University of Siena, Siena, Italy; 2 San Michele, Private Hospital, Albenga, Italy; 3 Department of Molecular and Developmental Medicine, University of Siena, Siena, Italy

## Abstract

**Background:**

Even if total knee replacement has revolutionized the treatment of degenerative knee diseases, the definition of an ideal rehabilitation protocol is still in progress. The aim of this study is to identify the factors that influence the outcomes after early, intensive, hospitalized treatment.

**Methods:**

A retrospective study was conducted in 2019 on 545 patients admitted to a northern Italy private clinic specialized in post-surgery rehabilitation, which applies a bio-psycho-social-environmental model and individual rehabilitation plans. Data regarding each patient were collected from medical records: age, numeric pain rating scale (NPRS) at admission, days between surgery and the beginning of rehabilitation (DBS). The outcomes were measured as the difference (Δ) between the values at discharge and admission of the Barthel scale (ΔBS), Tinetti scale (ΔTS), passive flexion (ΔPF) and active flexion (ΔAF). We performed a univariate linear regression through STATA, to determine which factors influence the outcomes. A p < 0.05 was considered statistically significant.

**Results:**

Our sample (69.17% female) was 69.8 ± 9.4 years old. ΔBS was significantly influenced by age (Coef. 0.270, CI [0.173 - 0.367]) and NPRS (Coef. 1.434, CI [0.979 - 1.890]). ΔTS was significantly influenced by age (Coef. 0.024, CI [0.011 - 0.037]) and NPRS (Coef. 0.130, CI [0.067 - 0.191]). ΔPF was significantly influenced by age (Coef. - 0.158, CI [- 0.266 - - 0.050]), DBS (Coef. - 1.047, CI [- 1.401 - - 0.687]) and NPRS (Coef. 1.825, CI [1.333 - 2.318]). ΔAF was significantly influenced by age (Coef. - 0.171, CI [- 0.300 - - 0.042]), DBS (Coef. - 1.150, CI [- 1.580 - - 0.721]) and NPRS (Coef. 2.504, CI [1.928 - 3.080]).

**Conclusions:**

Older patients obtain a higher functional outcome (ΔBS, ΔTS) but lower improvement in range of motion (ΔPF, ΔAF). Patients with higher NPRS at admission obtain an overall better outcome. Higher DBS is associated to lower articular outcomes (ΔPF, ΔAF).

**Key messages:**

• A better outcome can be achieved if rehabilitation is started immediately after knee replacement.

• Pain and age are not factors that impede effective rehabilitation.

